# Local Microscopical Societies

**Published:** 1881-01

**Authors:** Geo. E. Blackham


### LOCAL MICROSCOPICAL SOCIETIES.

### ORGANIZATION AND METHODS.

GEO. E. BLACKHAM, M. D., F. R. M. S.

The formation of the American Society of Microscopists has done much to advance the cause of Microscopy on this side of the Atlantic, and in no direction has its beneficial influence been more marked than in the impetus imparted to the formation of Local Microscopical Societies, a large number of which have been formed during the past two years.

In view of the number of these new organizations, it becomes pertinent to enquire, "How shall the benefits promised by such organizations be most fully secured?"

While the answer to this question must vary somewhat with circumstances, there is no doubt that thorough organization and a settled plan of work is almost absolutely necessary in all cases.

Under the head of organizations we may consider,

1st, *Membership.* Like most other questions this has two sides to it.

On the one hand it is urged that local societies should be composed exclusively of *workers* with as little "dead timber" as possible. The advocates of this plan say, in effect, that if the doors are thrown open and microscopists not engaged in original research admitted to membership, the standard of the society will be reduced and its meetings will be given over to mere display of "pretty slides and vainglorious comparisons of objectives or apparatus."

On the other hand it is urged that any one possessed of or having the use of a microscope should be welcome, and the list of members be made as large as possible.

The advocates of this plan say that the work of a society can not be averaged—that the work of "*workers*" will be none the worse because there may be many members incapable of doing the like and some incapable of appreciating it. That the beginners and amateurs will add to the interest of the meetings by furnishing an intelligent audience for the communications of their more advanced or more industrious fellow members, that they will form a preparatory class, from which it will be reasonable to expect that genuine workers will in time be developed, and that finally the membership fees and dues paid by this class of members will aid materially in placing the finances of the society in good condition, rendering the acquirement of suitable rooms, a cabinet and library possible in many cases where it would not be possible were the membership to be confined to the three or four "workers" who generally constitute (at first, at least,) the real scientific nucleous of a local microscopical society.

2d. Organization. This had better be as simple as possible. The less elaborate the Constitution and By-Laws, the better.

The officers should not be numerous.

A President, a Vice-President, a Secretary and a Treasurer are ample. The most important office is that of Secretary, and for it should be selected the most energetic and enthusiastic member of the society.

Until the society has grown old and large, it is best not to subdivide it into sections. When these are needed they can elect their own officers.

*Meetings.* The question of how often meetings should be held will often be affected by circumstances, but, as a rule, one meeting per month, from September to May inclusive, will be found sufficient. In a small society it would be difficult to keep up the interest, if meetings were held more frequently.

A good plan, adopted by some societies, is to leave all routine business, such as election of new members, payment of bills, &c., &c., in the hands of an executive committee, consisting of the officers of the society and two or more members elected for the purpose. This Committee transacts all ordinary routine business, and reports to the society annually or oftener, and thus relieves the regular meetings of the society from the tedious task of reading minutes and voting upon various motions and resolutions, and leaves the time free for the more important business of scientific discussion.

The frequency and date of the regular meetings having been determined upon, it is well to lay out in advance, each season, the work for the season, appointing a member to read a paper at each meeting, and as soon as possible, a card should be issued, giving on one side the list of officers and place of meeting, and on the other side a list of meetings for the coming season, giving the date of meeting, name of the essayist and the subject of the paper for each evening. This plan provides matter of interest for each meeting, gives time for proper preparation and enables members to prepare in advance to take any intelligent part in the principal discussion of the evening.

The order of business should be something as follows:

1st. Reading of stated paper.

2d. Discussion of same.

3d. Miscellaneous matters.

4th. Presentation and discussion of new instruments and accessories.

5th. Adjournment.

6th. Exhibition of objects and social hour.

If individual members can be induced to take up special branches and investigate them with great thoroughness, it will not only add greatly to the interest of the meetings and tend to keep the individual members at work by giving them a specific object to work for, but will, in addition, render it possible to make some genuine contributions to the stock of scientific knowledge.

The mere classification of the microscopic flora and fauna of any locality presents a rich and attractive field for a microscopical society to work up. Let one member take the diatoms of his immediate neighborhood, another the desmids, another the rhizopods, &c. After the lists have been completed, the life histories of individual species will afford delightful and useful work for years.

Regular reports of meetings should be published in the various microscopical and scientific journals, and members should be encouraged to take, at least, one of the journals in which these reports appear.

This brings up the question of a society library and cabinet. These every society should have, but in order to get their full benefits, should have also a suitable room to keep them in, which room could also be used for the meetings of the society.

Here the advantage of a large membership becomes apparent, for, by distributing the expense among many, it is made light for each one.

Assuming then that a room has been secured and properly furnished, the library should be begun by subscriptions to the leading microscopical journals, and also to those which, though not devoted exclusively to microscopy, have a microscopical department.

In addition, such standard and costly works of reference as "The Micrographic Dictionary," "Pritchard's Infusoria," "Wood's American Algæ," "Leidy's Rhizopods," &c., should be purchased from time to time as the finances of the society will permit. The cabinet should consist of slides and material purchased by the society or contributed by members or friends. The cheapest and most convenient way to keep them is in the white-wood racked boxes, hold ing twenty-five slides each. By means of a little paste and a strip of strong drilling, one of these boxes can be provided with a good hinged cover. When filled with slides they can be set up in shelves in rows like books and labelled. The slides lie flat, and a box containing twenty-five slides of any special class desired can be taken out without disturbing any others.

When the cabinet is once fairly started it will soon begin to fill up by contributions from members and friends, and it ought to be a part of the unwritten, if not of the written law, of the society that each member should contribute at least one well mounted slide per year to the cabinet. The slides to be preferably the personal work of the donor, but if this be not possible or convenient, then the work of some competent preparer. As the slides in the society cabinet will be used for comparison for identification of specimens, great care should be taken that they are *correctly* labelled and classified.

With such an organization as I have here sketched, with the serious workers each pursuing his chosen specialty, the amateurs contributing their time, their presence and their means, with commodious and attractive rooms centrally located, and containing a valuable and growing library and cabinet, a local microscopical society would soon become a centre of social and scientific influence, spreading a taste for scientific study, and making original and valuable contributions to the stock of scientific knowledge.

Without some such organization, the work will be purposeless and desulotry, interest will soon die out and the society die of inanition, without making any contribution to knowledge, or being of any special advantage to any one.

